# Comparing Perioperative Outcomes of Total Intravenous Anesthesia (TIVA) With Volatile Anesthesia in Patients With Obesity: A Systematic Review

**DOI:** 10.7759/cureus.54094

**Published:** 2024-02-12

**Authors:** Faiza A Kamal, Lucas Y Fernet, Naofal K Da Silva, Gabriela Briceño, Nusrath Iyoob, Kenneth Aleman Paredes, Marily Martinez Ramirez, Victor S Arruarana

**Affiliations:** 1 General Practice, University of Nottingham, Nottingham, GBR; 2 Surgery, Baptist Hospital of Miami, Miami, USA; 3 Obstetrics and Gynecology, Universidad de Oriente Núcleo de Anzoátegui, Barcelona, VEN; 4 Internal Medicine, Vinnytsia National Pirogov Medical University, Vinnytsya, UKR; 5 Surgery, Hospital General Regional IMSS (Instituto Mexicano del Seguro Social) No. 220 "General José Vicente Villada", Toluca, MEX; 6 Internal Medicine, Universidad Nacional Autonoma de Mexico, Mexico City, MEX; 7 Internal Medicine, Brookdale University Hospital Medical Center, New York City, USA

**Keywords:** volatile anesthesia, total intravenous anesthesia, recovery times, postoperative nausea and vomiting, obesity, intraoperative vital signs, inhalational anesthesia, emergence from anesthesia

## Abstract

In this systematic review, the perioperative outcomes of total intravenous anesthesia (TIVA) and volatile anesthesia were compared in obese adults (BMI ≥ 30 kg/m²) undergoing elective surgery. The review analyzed data from 12 randomized-controlled trials involving 935 patients, sourced from PubMed/MEDLINE (Medical Literature Analysis and Retrieval System Online), Cochrane, Scopus, and Web of Science databases. The focus was on intraoperative vital signs, emergence time, postoperative nausea and vomiting (PONV), duration of post-anesthesia care unit (PACU) stay, and ICU admission rates. Findings showed that TIVA (using propofol) might reduce PONV, but there were no significant differences in other outcomes compared to volatile anesthesia (with desflurane as the most common agent). The review highlights the need for more research, especially comparing sevoflurane with TIVA, to establish clear clinical guidelines for anesthesia in obese patients.

## Introduction and background

Obesity poses a significant risk for the development of heart disease, stroke, diabetes, and cancer, which are among the top 10 leading causes of death in the United Kingdom, the United States, and Canada [1-5]. It is defined as a body mass index (BMI) ≥ 30 kgm^2^. Global projections anticipate a rise in obesity from around 13% in 2016 [6] to 23-27% by 2035 [7]. The escalating incidence of obesity has led to a proportional increase in bariatric surgeries [8], posing greater challenges to anesthesiologists in the perioperative period [9]. 

Obesity is associated with increased incidence and severity of postoperative complications [10]. It results in an increase in both fat and lean mass; however, the increase in lean mass is considerably lower than fat, affecting the apparent volume of distribution of lipid-soluble anesthetic drugs [1]. Thus, the perioperative anesthetic strategy must be meticulously planned to address the altered physiology and pharmacokinetics in patients with obesity [11]. 

General anesthesia (GA) is commonly administered in one of two ways: balanced anesthesia utilizing a combination of intravenous and inhaled agents, or total intravenous anesthesia (TIVA), typically propofol-based, which only uses intravenous anesthetic agents for both induction and maintenance [12]. Existing comparisons between these techniques in patients with obesity are limited and lack consensus [13,14]. 

Propofol is an ideal drug for TIVA, given its rapid onset and short duration of action on the gamma-aminobutyric acid (GABA) receptors, altering cellular communication [15]. Although it is highly lipophilic, propofol has not consistently demonstrated prolonged effects of anesthesia in patients with obesity. This is potentially because the lipophilic properties of propofol are offset by the decreased perfusion of adipose tissue [16]. Similarly, during routine operations, the newer volatile anesthetic agents (desflurane, sevoflurane) have not demonstrated an increase in anesthetic time in patients with obesity compared to those with a normal BMI [16]. 

The aim of this systematic review was to compare the perioperative outcomes of volatile anesthetics and those with TIVA, specifically in patients with obesity undergoing elective surgery. The outcomes of interest in this review were evaluating intraoperative vital signs, time to emerge from anesthesia, the incidence of postoperative nausea and vomiting (PONV), length of stay in the post-anesthesia care unit (PACU), rates of admission to the ICU and reasons for admission to the ICU. Increased incidence of PONV can lead to delayed recovery and extended hospital stays [17], thereby increasing the risk of patients contracting hospital-acquired illnesses. Additionally, a lower incidence of PONV is likely to reduce PACU and hospital stay times, thereby having a positive impact on healthcare costs and effective resource utilization [17,18]. Recognizing the scarcity of literature in this area, an updated review was indicated to address the rising challenges presented in the perioperative care of patients with obesity.

## Review

Methods

As the first comprehensive review in this area, our primary objective was to map and analyze the existing literature. This approach helped identify research gaps and assess the feasibility of future meta-analyses. We conducted this systematic review using the criteria outlined in the Cochrane Handbook for Systematic Reviews of Interventions [19] and reported it according to the Preferred Reporting Items for Systematic Reviews and Meta-Analyses (PRISMA) 2020 guidelines [20,21]. This systematic review was registered with the International Prospective Register of Systematic Reviews (PROSPERO) (registration number: CRD42023491794).

Search Strategy

We searched PubMed/MEDLINE (Medical Literature Analysis and Retrieval System Online) (Table 1), Scopus (Table 2), Cochrane (Table 3), and Web of Science (Table 4) databases using Medical Subject Headings (MeSH) and free-text terms related to our study on 05/11/2023.

**Table 1:** Search on PubMed

Search	Results
((intravenous anesthesia[MeSH Terms]) OR (Intrevenous anesthesia[Title/Abstract]) OR (TIVA[Title/Abstract]) OR (Total Intravenous Anesthesia[Title/Abstract]) OR (Total Intravenous Anes*[Title/Abstract]) OR (fentanyl[Title/Abstract]) OR (sufentanil[Title/Abstract]) OR (remifentanil[Title/Abstract]) OR (iv anesthesia[Title/Abstract]) OR (propofol[Title/Abstract]) OR (anaesthetics intravenous[Title/Abstract])) AND ((inhalation anesthesia[MeSH Terms]) OR (inhalation anaesthetics[MeSH Terms]) OR (inhalation anesthesia[Title/Abstract]) OR (inhalation anaesthetics[Title/Abstract]) OR (Volatile Anesthesia[Title/Abstract]) OR (desflurane[Title/Abstract]) OR (sevoflurane[Title/Abstract]) OR (isoflurane[Title/Abstract]) OR (volatile anaesthetics[Title/Abstract]) OR (inhalational anesthesia[Title/Abstract]) OR (inhaled anesthesia[Title/Abstract])) AND ((obesity[MeSH Terms]) OR (morbid obesities[MeSH Terms]) OR (obesity[Title/Abstract]) OR (morbid obesities[Title/Abstract]) OR (Obese[Title/Abstract]) OR (Severely Obese[Title/Abstract]))	129

**Table 2:** Search in Scopus

Search	Results
intravenous AND anesthesia AND volatile AND anesthesia AND obesity	697

**Table 3:** Search in Cochrane

Search	Results
#1 MeSH descriptor: [Anesthesia, Intravenous] explode all trees	2036
#2 (TIVA):ti,ab,kw	1101
#3 (Total Intravenous Anesthesia):ti,ab,kw	6484
#4 ("total intravenous anesthesia"):ti,ab,kw	1742
#5 (Total Intravenous Anes*):ti,ab,kw	6716
#6 (fentanyl):ti,ab,kw	17581
#7 (sufentanil):ti,ab,kw	3943
#8 (remifentanil):ti,ab,kw	5949
#9 (iv anesthesia):ti,ab,kw	10761
#10 (propofol):ti,ab,kw	18116
#11 (anaesthetics intravenous):ti,ab,kw	7601
#12 MeSH descriptor: [Anesthesia, Inhalation] explode all trees	1999
#13 MeSH descriptor: [Anesthetics, Inhalation] explode all trees	2805
#14 (inhalation anesthesia):ti,ab,kw	5830
#15 (inhalation anaesthetics):ti,ab,kw	3561
#16 (Volatile Anesthesia):ti,ab,kw	1182
#17 (desflurane):ti,ab,kw	1923
#18 (sevoflurane):ti,ab,kw	6893
#19 (isoflurane):ti,ab,kw	4411
#20 (volatile anaesthetics):ti,ab,kw	912
#21 (inhalational anesthesia):ti,ab,kw	2018
#22 (inhaled anesthesia):ti,ab,kw	917
#23 MeSH descriptor: [Obesity] explode all trees	21528
#24 MeSH descriptor: [Obesity] explode all trees	21528
#25 (obes*):ti,ab,kw	54401
#26 (morbid obes*):ti,ab,kw	2948
#27 (Severely Obese):ti,ab,kw	418
#28 (#1 OR #2 OR #3 OR #4 #5 OR #6 OR #7 OR #8 OR #9 #10 OR #11) AND (#12 OR #13 OR #14 OR #15 OR #16 #17 OR #18 OR #19 OR #20 #21 OR #22) AND (#23 OR #24 OR #25 OR #26 OR #27)	124

**Table 4:** Search in Web Of Science

Search	Results
1: All=Anesthesia, Intravenous	21341
2: All=TIVA	1279
3: All=Total Intravenous Anesthesia	6018
4: All=Total Intravenous Anes*	8172
5: All=fentanyl	23596
6: All=sufentanil	4325
7: All=remifentanil	7592
8: All=iv anesthesia	7274
9: All=propofol	32069
10: All=anaesthetics intravenous	11035
11: #10 OR #9 OR #8 OR #7 OR #6 OR #5 OR #4 OR #3 OR #2 OR #1	78225
12: All=Anesthesia, Inhalation	4305
13: All=Anesthetics, Inhalation	3431
14: All=inhalation anesthesia	4305
15: All=Volatile Anesthesia	4912
16: All=desflurane	3959
17: All=sevoflurane	13888
18: All=isoflurane	20317
19: All=volatile anaesthetics	8109
20: All=inhalational anesthesia	1814
21: All=inhaled anesthesia	2973
22: #12 OR #13 OR #14 OR #15 OR #16 OR #17 OR #18 OR #19 OR #20 OR #21	38027
23: All=obesity	500962
24: All=obes*	606051
25: All=obese	185753
26: #23 OR #24 OR #25	606051
27: #26 AND #22 AND #11	170

Types of Study

Strict inclusion and exclusion criteria were used to identify robust studies for analysis. The aim of this study was to conduct a systematic review of relevant studies available in English, with no constraints on the date of publication. We methodically screened and studied randomized controlled trials (RCTs). The studies that were chosen to be included in this review met the following inclusion criteria: RCTs comparing the effects of different types of GA; either a combination of volatile and intravenous agents, or TIVA. We excluded case reports, case series, case-control, cohort studies, comment publications, book chapters, protocol articles, reviews, dissertations, letters to the editor, editorials, news articles, and pro-con debates. Additionally, we excluded research articles that were not available in full text or could not be accessed through interlibrary loans, as well as ones that did not provide a clear description of their operationalization.

Types of Participants

This review had clear participant selection criteria. Adults (patients over the age of 18 years) of all genders and ethnicities, who had a BMI ≥ 30 kgm^2^, and had undergone elective surgery under GA, were included in the study. Studies reporting on the outcomes of using GA in the pediatric population, in animals, or in patients with a BMI of < 30 kgm^2^ were excluded. Additionally, patients undergoing emergency surgery were excluded as the morbidity and mortality in these patients can be unpredictable.

Types of Intervention and Comparator

Articles included in this study must have reported on the intra- and postoperative effects of GA used in adult patients with obesity. The intervention group would be patients who received TIVA. The comparator would be patients who received a combination of intravenous and volatile anesthetic agents. Studies reporting on patients who only received sedation or local/regional anesthesia were excluded. 

Outcomes

The outcomes of interest in this review were intraoperative vital signs (heart rate, respiratory rate, blood pressure, mean arterial pressure, and oxygen saturation), time to emerge from anesthesia (measured as the time to tracheal extubation, time to eye opening, Aldrete score, or other specified measures), the incidence of PONV, length of stay in the PACU, rates of admission to the ICU, and reasons for admission to the ICU. Any studies that did not report on at least one of these outcomes were excluded.

Selection of Studies and Extraction of Data

In an initial screening based on the title and abstract, two reviewers (ECM and NDS) independently selected trials for inclusion in this review using predetermined inclusion and exclusion criteria. This search was performed using Rayyan [22] to extract relevant data, and duplicates were filtered. Keywords were employed to highlight inclusion and exclusion criteria-related words on Rayyan [22]. Any disagreements about the inclusion of studies were resolved by consulting a third review author (FAK). 

Subsequently, a full-text analysis was conducted, with two reviewers (GBS and LYF) independently selecting trials for inclusion in this review using predetermined inclusion and exclusion criteria. Disagreements about the inclusion of studies were resolved by consulting a third review author (FAK).

Data Evaluation: Assessment of Risk of Bias in Included Studies

To assess the quality of studies included in the systematic review, we applied the Cochrane Risk of Bias 2.0 Tool [23] for RCTs. Two independent reviewers evaluated the risk of bias in each study (LYF and NMI), considering the specific criteria and guidelines provided by the respective tools. Any discrepancies between the reviewers were resolved by consulting with a third, blinded reviewer (ECM). The methodological components of the trials were assessed as having some concerns, a low or high risk of bias in accordance with the Cochrane Handbook [19]. 

Results

Study Identification and Selection

**Figure 1:**
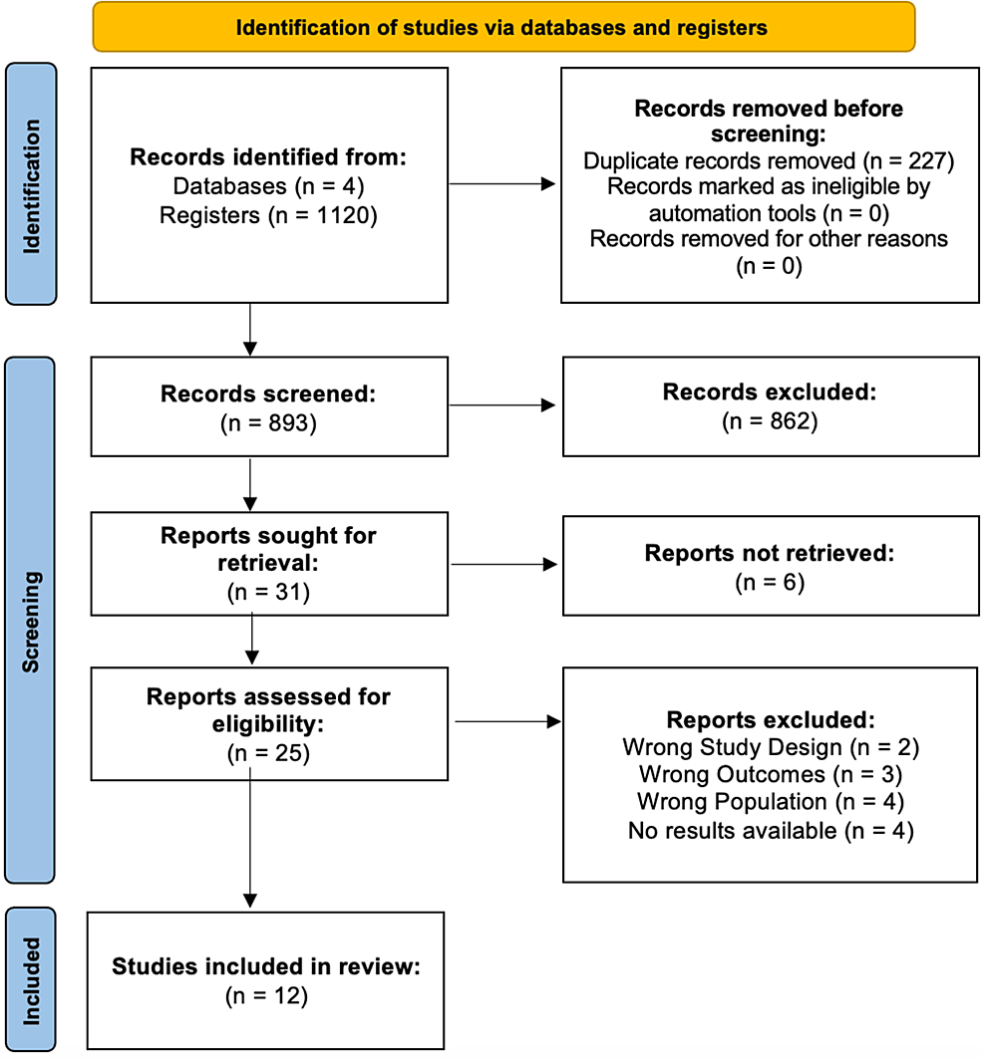
PRISMA flowchart Summary of the process of selection of studies included in this systematic review.

**Figure 2:**
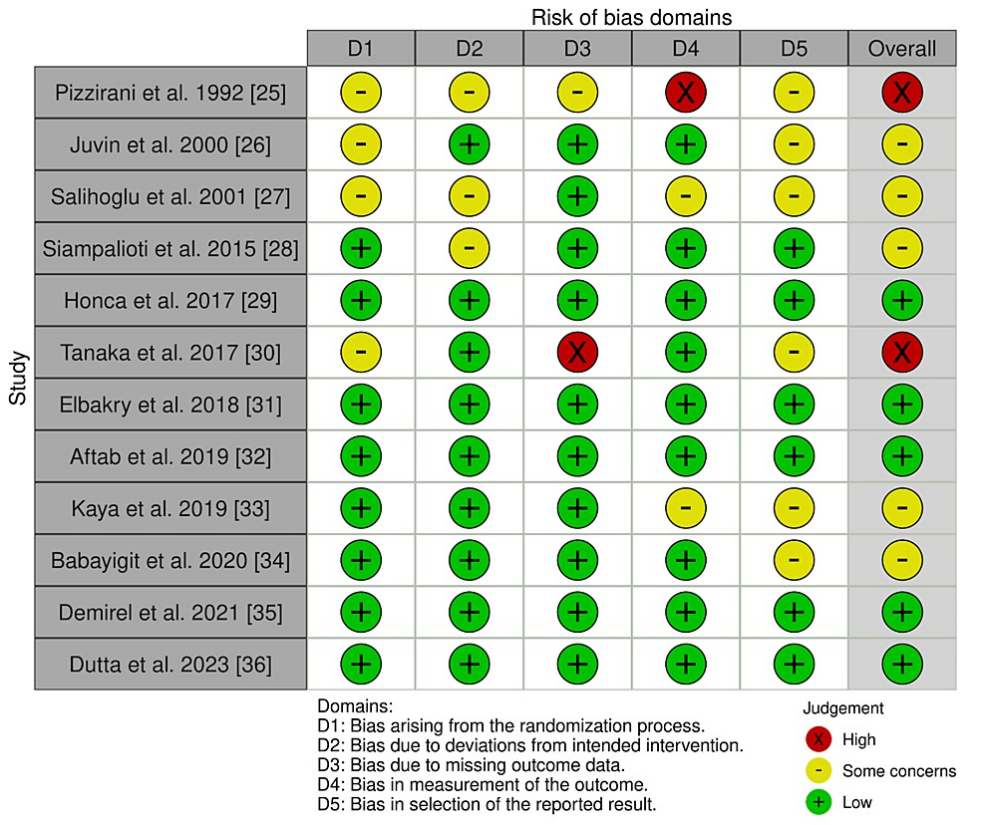
Risk-of-bias analysis using robvis Summary of the risk-of-bias analysis of the included studies, demonstrating low risk (green), some concerns (yellow), and high risk for bias (red) [24-36] ​​​​ robis: risk-of-bias visualization, an R package and web application for visualizing risk-of-bias assessments (https://github.com/mcguinlu/robvis)

Across the databases, we were able to narrow the pool of possible articles down to 1120 articles. After a thorough examination, 227 duplicate articles were eliminated. Following the screening of the titles and abstracts of 893 articles, 31 publications were selected for full-text screening. Their eligibility and quality was assessed and 12 studies were selected for inclusion in this review. Figure 1 shows the PRISMA [20] flowchart depicting the studies' selection procedure, and Figure 2 shows the assessment of the risk of bias of articles using the appropriate Cochrane Risk-of-Bias tool 2.0 [23].

Synthesis of the Results

Study characteristics: The included studies were prospective RCTs primarily focusing on bariatric surgeries, except one study which evaluated patients undergoing total knee replacement (Table 5) [30]. Propofol served as the primary TIVA agent, while inhalational anesthesia involved either desflurane (with propofol induction) [26,29-32,35,36], sevoflurane [28,33,34], sevoflurane (with sevoflurane induction using single breath technique) [27], or isoflurane (with thiopental induction) [25]. 

**Table 5:** General outcomes of included studies RCT: Randomized-Controlled Trial, BMI: Body Mass Index, BIS: Bispectral Index, TIVA: Total Intravenous Anesthesia, PSI: Patient State Index, MAP: Mean Arterial Pressure, PONV: Postoperative Nausea and Vomiting, PACU: Post-Anesthesia Care Unit * Relevant to the measured outcomes of this systematic review. ^# ^At least 17 patients were excluded from the results for various reasons which included PONV, oversedation and postoperative pain, however, as this was not their primary measured outcome, it was not reported which group these patients belonged to.

Author and year	Country	Type of Study	Population Characteristics	Number of Patients	Intervention and Comparator	Results *
Pizzirani et al., 1992 [25]	Italy	Prospective, non-blind RCT	Adults (17-60 years) with class III obesity (measured in percentage of ideal body weight) undergoing elective restrictive gastric surgery	40	Group 1: TIVA (propofol) Group 2: Isoflurane with thiopental	There were no significant differences in the modified Aldrete scores between the two groups.The recovery time was shorter in the isoflurane group compared to the TIVA group, however, this difference showed a low degree of statistical significance (0.05 < p < 0.1).
Juvin et al., 2000 [26].	France	Prospective, double-blind RCT	Adults (mean age 39) with BMI > 35 undergoing laparoscopic gastroplasty	36	Group 1: Desflurane; Group 2: TIVA (propofol); Group 3: Isoflurane	Time to extubation, eye opening, and orientation was significantly reduced in the desflurane group compared to the other two groups (p < 0.05). SpO_2_ values and mobility at PACU admission were significantly improved in the desflurane group compared to the other two groups (p < 0.05). Sedation was significantly less pronounced in the desflurane group at 30 and 120 mins postoperatively compared to the other two groups (p < 0.05). There were no significant differences in PONV or length of PACU stay. One patient in the isoflurane group experienced bronchospasm after eye opening, requiring admission to ICU.
Salihoglu et al., 2001 [27]	Turkey	Prospective RCT	Adults (19-63 years) with BMI > 35 undergoing stoma-regulated gastric banding	40	Group 1: TIVA (propofol) Group 2: Sevoflurane	Intraoperative and early postoperative MAP values were significantly lower in the TIVA group (p < 0.05). There were no statistically significant differences between the groups regarding heart rates and SpO_2_. There was no significant difference in eye opening in response to verbal stimulus or in time taken for the patient to orientate to time, place, and person.
Siampalioti et al., 2015 [28]	Greece	Prospective, double-blind RCT	Adults (21-60 years) with BMI > 50 undergoing elective bariatric surgery	100	Group 1: TIVA (propofol) without BIS monitoring; Group 2: TIVA (propofol) with BIS Group 3: Sevoflurane without BIS Group 4: Sevoflurane with BIS	No significant differences in heart rates between the four groups. Intraoperative MAP was higher in both TIVA groups (p < 0.001). Time to emerge from anesthesia was significantly shorter in both TIVA groups (p < 0.001). Time to extubation was significantly shorter in both TIVA groups (p < 0.001). Recovery measured using Aldrete score was not significantly different in all four groups but using Chung and White scores, recovery was shorter in both sevoflurane groups (p = 0.001 and p = 0.002, respectively).
Honca et al., 2017 [29]	Turkey	Prospective, double-blind RCT	Adults (mean age 38) with BMI > 35 undergoing laparoscopic sleeve gastrectomy	61	Group 1: TIVA (propofol); Group 2: Desflurane	There were no statistically significant differences in intraoperative vital signs between the two groups. Time to eye opening and extubation were significantly shorter with desflurane (p < 0.05). There were no significant differences in recovery scores, measured using the Aldrete score. One patient in the desflurane group experienced bronchospasm during extubation, requiring admission to ICU.
Tanaka et al., 2017 [30]	United States	Prospective, double-blind RCT	Adults (> 65 years) with BMI > 30 undergoing elective total knee arthroplasty	100	Group 1: TIVA (propofol); Group 2: Desflurane	There were no significant differences reported in time to emerge from anesthesia (time to eye opening and to extubation), incidence of PONV^# ^or length of PACU stay.
Elbakry et al., 2018 [31]	Egypt	Prospective, double-blind RCT	Adults (30-50 years) with class III obesity undergoing laparoscopic sleeve gastrectomy	100	Group 1: Desflurane; Group 2: TIVA (propofol)	TIVA group consistently had lower intraoperative heart rates and MAP (p < 0.0001). There was no significant difference between the two groups regarding time to recover from anesthesia, measured using the Aldrete score. TIVA group had significantly lower incidence of postoperative nausea (p = 0.01) and vomiting (p = 0.03), and shorter PACU stay time (p = 0.01) as compared with desflurane.
Aftab et al., 2019 [32]	Norway	Prospective, double-blind RCT	Adults (mean age 44) with BMI > 35 undergoing elective laparoscopic sleeve gastrectomy or Roux-en-Y gastric bypass	183	Group 1: TIVA (propofol); Group 2: Desflurane	No significant differences were found in visual analogue scale scores for nausea and vomiting postoperatively. There was no difference in the time to awakening between the two study groups.
Kaya et al., 2019 [33]	Turkey	Prospective, single-blind RCT	Adults (18-65 years) with BMI > 35 undergoing laparoscopic sleeve gastrectomy	60	Group 1: TIVA (propofol); Group 2: Sevoflurane	No significant difference in intraoperative MAP was found. The difference in heart rates and oxygen saturations between the two groups were not consistently statistically significant. Recovery time was significantly longer in the TIVA group compared to the sevoflurane group (p < 0.001).
Babayigit et al., 2020 [34].	Turkey	Prospective, double-blind RCT	Adults (18 to 65 years) scheduled to undergo bariatric surgery	55	Group 1: TIVA (propofol) Group 2: Sevoflurane	No significant differences in MAP or heart rate were found between the TIVA and sevoflurane groups.
Demirel et al., 2021 [35].	Turkey	Prospective, double-blind RCT	Adults (21 to 60 years) with BMI > 40 undergoing elective sleeve gastrectomy	120	Group 1: TIVA (propofol) with PSI monitoring; Group 2: TIVA (propofol) without PSI; Group 3: Desflurane with PSI; Group 4: Desflurane without PSI	No significant differences were found in duration of anesthesia, admittance to PACU, discharge from PACU or in the modified Aldrete score. There were no differences in the perioperative blood pressure or heart rate amongst all four groups. PONV scores were significantly lower in both TIVA groups and in the desflurane-PSI group compared to desflurane without PSI. Recovery durations in both TIVA groups were significantly lower than the desflurane without PSI group (p = 0.02).
Dutta et al., 2023 [36]	India	Prospective, double-blind RCT	Adults (18-65 years) with BMI > 35 undergoing elective laparoscopic bariatric surgery	40	Group 1: TIVA (propofol) Group 2: Desflurane	No statistically significant difference was found in time-to-eye opening, time-to-tracheal extubation or ability-to-shift score from operating table to transport bed. There was no significant difference in time to achieve a modified Aldrete score > 9/10. No significant difference in intraoperative heart rate or MAP was found. However, following extubation, the heart rate was significantly higher in the desflurane group (p < 0.05). There was no significant difference in the postoperative sedation score, or in the visual analogue scores for incidence of PONV in the first 24 hours post-surgery.

Propofol doses varied, administered based on either the corrected body weight (CBW) as described by Servin and colleagues [37] or on the ideal body weight (IBW). One study used a patented closed-loop anesthesia delivery system (CLADS) for TIVA dosing [36]. Induction with propofol in this study was based on lean body weight, whereas maintenance with propofol was based on adjusted body weight. The automated administration of propofol by the CLADS system was programmed to maintain a target bispectral index (BIS) value of 50. The inhalational anesthesia maintenance dosing varied considerably between studies.

Risk of bias: As shown in Figure 2, five studies had a low risk of bias, with no concerns in any of the domains regarding their process of randomization, deviations from the intended intervention, bias due to missing outcome data or its measurement, or bias in selection of the reported results [29,31,32,35,36]. Another five studies had some concerns of bias overall in their methodology [26-28,33,34]. Lastly, two studies were deemed to have an overall high risk of bias in their methodology. In one study, this was due to concerns in measurement of the outcome [25] and in the second study, it was because of missing outcome data from several patients [30].

Intraoperative Vital Signs

Eight studies reported on intraoperative vital signs. Four studies reported no statistically significant differences in the heart rates in a total of 336 patients [28,29,34,35]. One study reported that in all 100 patients studied, despite having comparable heart rates at baseline, the TIVA group recorded significantly lower heart rates from induction to discharge from PACU [31]. Another study reporting on 40 patients found no significant difference in the heart rates and non-invasive mean arterial pressure (MAP) between the two groups intraoperatively; however, when measured following extubation, reported heart rates were higher in the desflurane group [36]. A third study (60 patients) reported that the heart rates in the TIVA group were higher than in the volatile anesthesia (sevoflurane) group, with statistically significant values at baseline (preoperative measurement), at 10 minutes following induction and between 40 minutes and 75 minutes of operating time [33]. However, these statistically significant differences in heart rate did not occur consecutively. The other studies did not report intraoperative heart rates.

The intraoperative MAP was also variable between the different studies. Five studies (336 patients) reported no difference between the two groups [29,33-36], while one study (100 patients) reported higher MAP in patients who received TIVA [28] and two studies (140 patients) reported a lower MAP [27,31] in the TIVA group as compared with volatile anesthesia. Three articles reported on the SpO2 values, with two (101 patients) finding no difference between the groups [27,29], and one (60 patients) reporting a statistically significant drop in the SpO2 between 50 minutes and 60 minutes of operative time in the TIVA group [33]. The rest of the vital signs were not reported in the included studies.

Time to Emerge from Anesthesia

Eight studies (620 patients) reported on the time to emerge from anesthesia. The outcomes were variable. The time to extubation and/or time to eye opening was found to be significantly longer in the TIVA group in three studies (157 patients) [26,29,33], significantly shorter in the TIVA group in one (100 patients) [28], and no statistically significant differences were found in time to emerge from anesthesia between the TIVA and volatile group in four studies (363 patients) [27,30,32,36]. 

PONV

Of the five studies (396 patients) that reported PONV, three found no difference in the incidence of PONV (176 patients) [26,30,36], and two studies (220 patients) reported that patients who received TIVA had a lower incidence of these effects [31,35]. 

Length of Recovery and PACU Stay

Aldrete's scoring system [38] as well as the scoring systems developed by Chung [39] and White [40] were used to assess when postsurgical patients were ready to be discharged from PACU. Of the six studies (totaling 461 patients) that measured the Aldrete score, none found a statistically significant difference in the recorded scores [25,28,29,31,35,36]. Only one study (100 patients) measured Chung and White scores in addition to the Aldrete score and found these two scores to be significantly lower in the volatile (sevoflurane) anesthesia group [28]. Length of stay in the PACU was reported in three studies (256 patients); none of these demonstrated any significant difference between the use of TIVA or volatile anesthesia [26,30,35]. 

ICU Admissions

Only two studies reported a patient each who was admitted to the ICU, but these patients’ data were excluded from their analysis. Both reported patients who developed bronchospasm around the time of extubation, requiring sedation, mechanical ventilation, and subsequent admission to ICU [26,29]. Both patients were in the volatile anesthesia groups: desflurane [29] and isoflurane [26].

Discussion

This systematic review evaluated the intraoperative and postoperative outcomes of TIVA and volatile anesthesia, focusing on intraoperative vital signs, time to emerge from anesthesia, PONV, length of PACU stay, and rates of ICU admissions.

Overall, the studies that reported on the length of PACU stay and recovery scores (measured using the Aldrete score) consistently showed no significant difference between the two anesthetic regimens. While a trend emerged favoring TIVA in reducing the incidence of PONV from the studies evaluated in this review, other outcomes did not exhibit conclusive differences.

Intraoperative Vitals

There was limited literature reporting on intraoperative vital signs and the results of this review showed great variability, making it challenging to establish a superior anesthetic regimen. Significant heterogenicity in the effects of TIVA and volatile anesthetics on intraoperative heart rates and oxygen saturations suggest both anesthetic techniques are suitable in patients with obesity without increasing the risk of rate- or hypoxia-related complications.

Similarly, no consistent evidence emerged regarding the effects on intraoperative MAP, with considerable variability in reported results. A recent systematic review and meta-analysis reported that TIVA resulted in significantly decreased MAP [13]; however, this conclusion was based on only one study and, therefore, cannot establish a reliable association. A lack of definitive results may be attributable to the fact that blood pressure and heart rates are complex physiological parameters that are controlled by various centers in the body and display considerable variability between individuals. Moreover, while the hypotensive effects of both propofol and volatile anesthetic agents are well-documented in literature [41-44], substantial research directly comparing differences in physiological parameters between the two techniques is lacking. 

Emergence from Anesthesia

There was no clear superiority demonstrated between TIVA and volatile anesthesia regarding the time to emerge from anesthesia. It is worth noting that the eight studies that reported on this chose different variables to measure this outcome. Delayed emergence from anesthesia is multifactorial; residual action of several different agents can cause this, especially benzodiazepines, opioids, and propofol [45]. Inappropriate dosing with propofol is not uncommon when using open target-controlled infusion (TCI) systems to provide TIVA. Most TCI pumps predict plasma and effect site concentration of infusing drugs [46], extrapolating from algorithms based on populations from the 1990s which did not include patients of either extreme of weight [47,48]. Obesity can alter the pharmacokinetics of certain drugs due to the increase in adipose tissue, changes in lean body mass, alterations in cardiac output associated with obesity, and an increase in extracellular fluid volume [16,49]. The mismatch between the TCI pump algorithm and the physiological behaviors of patients with obesity can make it challenging for anesthesiologists to accurately dose weight-based drugs, leading to unpredictable anesthetic effects [50].

Alternative models for TCI pump algorithms are now being explored, including the Eleveld model, which accounts for extremes of weight [51]. Furthermore, due to its high lipid solubility, propofol may display unusual pharmacokinetics in patients with obesity, particularly during longer surgeries [52]. Volatile anesthetic agents also display variable degrees of lipid solubility but to a lesser degree than propofol [52]. A previous systematic review and meta-analysis comparing various volatile agents and propofol found that patients who had been under desflurane maintenance had a shorter time to eye opening, extubation, and stating their names compared to patients in isoflurane and propofol groups. The same study found no difference in time to emerge from anesthesia between propofol, isoflurane, and sevoflurane groups [13]. These findings were partially supported by another systematic review and meta-analysis which, although evaluating effects in non-obese patients, showed significantly shorter time to emerge from desflurane compared to propofol. It also reported a significantly shorter time to obey commands in sevoflurane compared to propofol, but no significant differences between isoflurane and propofol [53]. Variability in the results could also be due to several other confounding factors, such as differences in the combinations of drugs used, length of surgeries, anesthesiologists’ training and experience, and equipment available at different institutions. Consequently, it is difficult for strong recommendations to be made on the preferred anesthetic technique for faster emergence in patients with obesity. However, overall, there is some evidence that desflurane may deliver favorable outcomes in this population. 

Length of PACU Stay and Recovery Scores

All studies which reported on either the length of PACU stay or the Aldrete score to assess patients’ readiness to be discharged from PACU, found no significant difference comparing TIVA to volatile anesthetics. A previous systematic review of patients with obesity has reported similar results [13]. Interestingly, one study [28] in the present review found no difference when using the modified Aldrete score but reported significantly shorter recovery times in the sevoflurane groups when using the Chung and the White recovery scores. These scores differ from the modified Aldrete score in that they also evaluate PONV and postoperative pain. The study did not provide a clear explanation to address the inconsistencies in the results of the different scoring systems [28].

PONV

Out of the three studies that showed no significant differences in the incidence of PONV between TIVA and volatile anesthesia [26,30,36], two were deemed to have some concerns [26] or high risk for bias [30] (Figure 2). This was largely due to the number of patients that were excluded from their studies, possibly due to outcomes of interest such as severe PONV. On the other hand, the two studies [31,35] that reported a statistically significant decrease in PONV in the TIVA groups were among the studies that were deemed to be of high quality due to their low risk of bias in all domains and no missing participant data. Furthermore, these two studies [31,35] reported data from a much larger sample size than each of the other three studies [26,30,36], and therefore, their results were deemed to be more reliable than the others. This conclusion was corroborated by other recent systematic reviews and meta-analyses which compared outcomes with the use of TIVA and volatile anesthesia. One review found that there was a significantly reduced incidence of PONV in patients receiving TIVA [14]. This was further supported by another systematic review conducted in non-obese patients which found a significant reduction in rates of PONV in TIVA compared to isoflurane, desflurane, and sevoflurane [53]. Although conducted in pediatric patients, a further systematic review and meta-analysis found significantly lower incidences of PONV in patients who received TIVA [54]. Although another systematic review found no difference in PONV between TIVA and inhaled anesthesia, it is worth noting that this conclusion was formed after the evaluation of only one study [13]. Considering the breadth of evidence in favor of TIVA regarding reduced PONV, it can be reliably concluded that TIVA may be a better option for patients who are at an increased risk of PONV. While obesity itself is not a risk factor for PONV [55,56], it is associated with longer operating times. A 30-minute increase in operating time may increase the risk of PONV by 60% [57]. Although an individual approach is always recommended when considering the anesthetic options for each patient, patients who have a higher inherent risk of PONV, or are undergoing surgeries that are known to increase the risk of PONV (intraabdominal, gynecological, maxillofacial) [58], are likely to benefit from TIVA. 

ICU Admissions

Two patients were admitted to the ICU in two different studies, both due to bronchospasm occurring immediately following extubation [26,29]. It is worth noting that both patients were part of the inhalational anesthesia groups. However, there is insufficient evidence to suggest an association between volatile anesthesia and the incidence of ICU admission as there is no information about the possibility that this outcome was due to a register of blood transfusion or administration of other drugs [59]. 

Limitations

While this systematic review provides a comprehensive comparison between total intravenous and volatile anesthesia in patients with obesity, filling a critical gap in recent literature, there are some notable limitations. The majority of included studies compared TIVA with desflurane; only a small percentage drew comparisons between TIVA and sevoflurane. As more centers recognize the harmful environmental effects and significant cost of desflurane [60,61], and discontinue its use in regular clinical practice, the perceived benefits of desflurane over TIVA seem less relevant. Some of these benefits did, however, have an influence on the conclusions drawn in this study. Therefore, this review demonstrates the critical need for comprehensive trials comparing the effects of TIVA with sevoflurane in patients with obesity, in order to highlight potential differences which are more clinically relevant and useful. Additionally, this review exclusively retrieved articles published in English that were publicly available without institutional access. It is therefore possible that some high-quality research was inadvertently excluded. Furthermore, some registered trials meeting the inclusion criteria were excluded due to unreported results. Finally, despite evaluating more studies than previous comparable systematic reviews, the small sample size may limit the generalizability of the findings to a broader population of patients with obesity. Perhaps this could also be a reason why some studies found no significant differences in their investigated outcomes. With the rising incidence of obesity, larger, high-quality randomized-controlled trials are essential to establish more definitive conclusions and to better guide perioperative care to reduce complications.

## Conclusions

This systematic review compared perioperative outcomes of TIV with those of balanced anesthesia using volatile agents. While TIVA demonstrated a potential benefit in reducing the incidence of PONV, other outcomes did not show conclusive differences. The small number of available studies for evaluation, their small sample sizes of patients, and the scarcity of trials specifically investigating the effects of sevoflurane compared with TIVA, underscores the necessity for further research. As the prevalence of obesity continues to rise, future research endeavors in this field should prioritize larger, high-quality RCTs to refine anesthetic strategies and improve outcomes in this population. Guidelines can then be developed explaining the safest anesthesia techniques to meet their unique physiological needs, thereby allowing an improvement in the quality of patient care.
